# Supplementary material for: Minocycline Inhibits Tick-Borne Encephalitis Virus and Protects Infected Cells via Multiple Pathways
Source: Viruses. 2024 Jun 29;16(7):1055. doi: 10.3390/v16071055 (PMC11281541; doi:10.3390/v16071055)
Supplement: Supplementary file 1 [file viruses-16-01055-s001.zip › Supplementary Materials.pdf]

Table S1 list of specific primers

primers	suquences(5'-3')
TBEV-F	TGGGCGGTTCTTGTTCTCC
TBEV-R	TCACACATCACCTCCTTGTCAGA
TBEV-P	CTGAGCCACCATCACCCAGACACAG
PSMB4-F	CGGCCCCAGGACAGTTTT
PSMB4-R	GCGTGATTGGACCTCTGTAAAGT
PSMB4-P	CCGCATTCCGTCCACTCCCGATTCC
SNAP25-F	AGTGGACGAACGGGAGCAGAT
SNAP25-R	GTGACGGAGGTTCCCGATGAT
FGF2-F	TGTGCTAACCGTTACCTTGC
FGF2-R	CTGCCCAGTTCGTTTCAGTG
PDGFRA-F	TAGTGCTTGGTCGGGTCTTGG
PDGFRA-R	CAGGTTGGGACCGGCTTAATC
PLCB2-F	GCCGAGCAAATCTCCAAAATGA
PLCB2-R	GGTGTCGATCTCCGCCGTCT
STY11-F	GTGAACGTCTACTACGGCAGAAAGC
STY11-R	GCGGGGATGTCGTAGATGAAAG
CALML4-F	TGTTGATGGCGGACAAGGAGA
CALML4-R	CATACTTCACTTTGCCGTTGGGTT
RYR2-F	TGAGAATGAAACCCTTGACTATGAG
RYR2-R	GAAAGTCTGAAGTCGGGTATCGTTG
IL-6-F	ACAGCCAGCCACTGACCTCTTC
IL-6-R	CAGTGCCTCTTTGCTGCTTTCA
GADPH-F	AAATCAAGTGGGGCGATGCTG
GADPH-R	ATGATGACCCTTTTGGCTCCC

Table S2 fluorescent quantitative PCR reaction procedure of Taqman

Tempreture	Times	Cycles
50 ℃	15 min	1 cycle
95 ℃	3 min	1 cycle
94 ℃	10 s	45 cycles
58 ℃	45 s	

Table S3 fluorescent quantitative PCR reaction procedure of SYBR Green

Tempreture	Times	cycles
95 ℃	30 s	1 cycle
95 ℃	5 s	40 cycles
60 ℃	10 s	
65 ℃	5 s	1 cycle
95 ℃	0 s	1 cycle

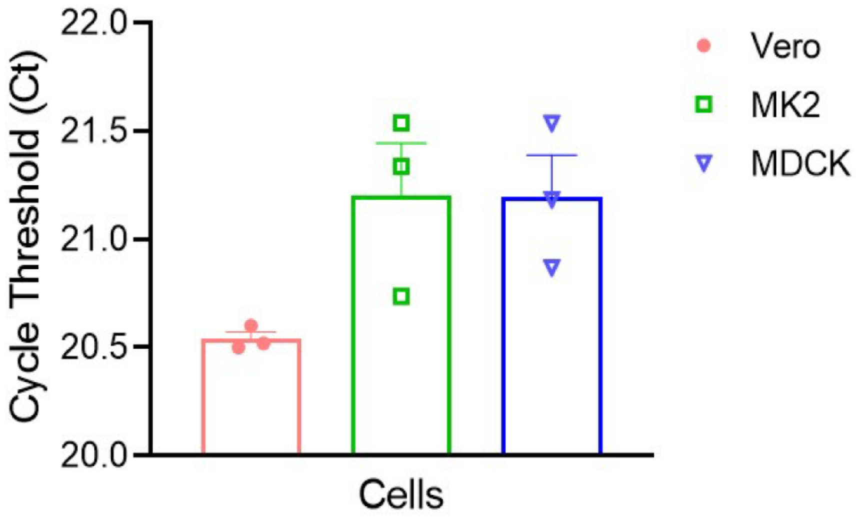

Figure S1. **The level of TBEV reproduction in different cells**: The Ct value of TBEV was cultured in Vero, MK2 and MDCK cells were detected b RT-qPCR

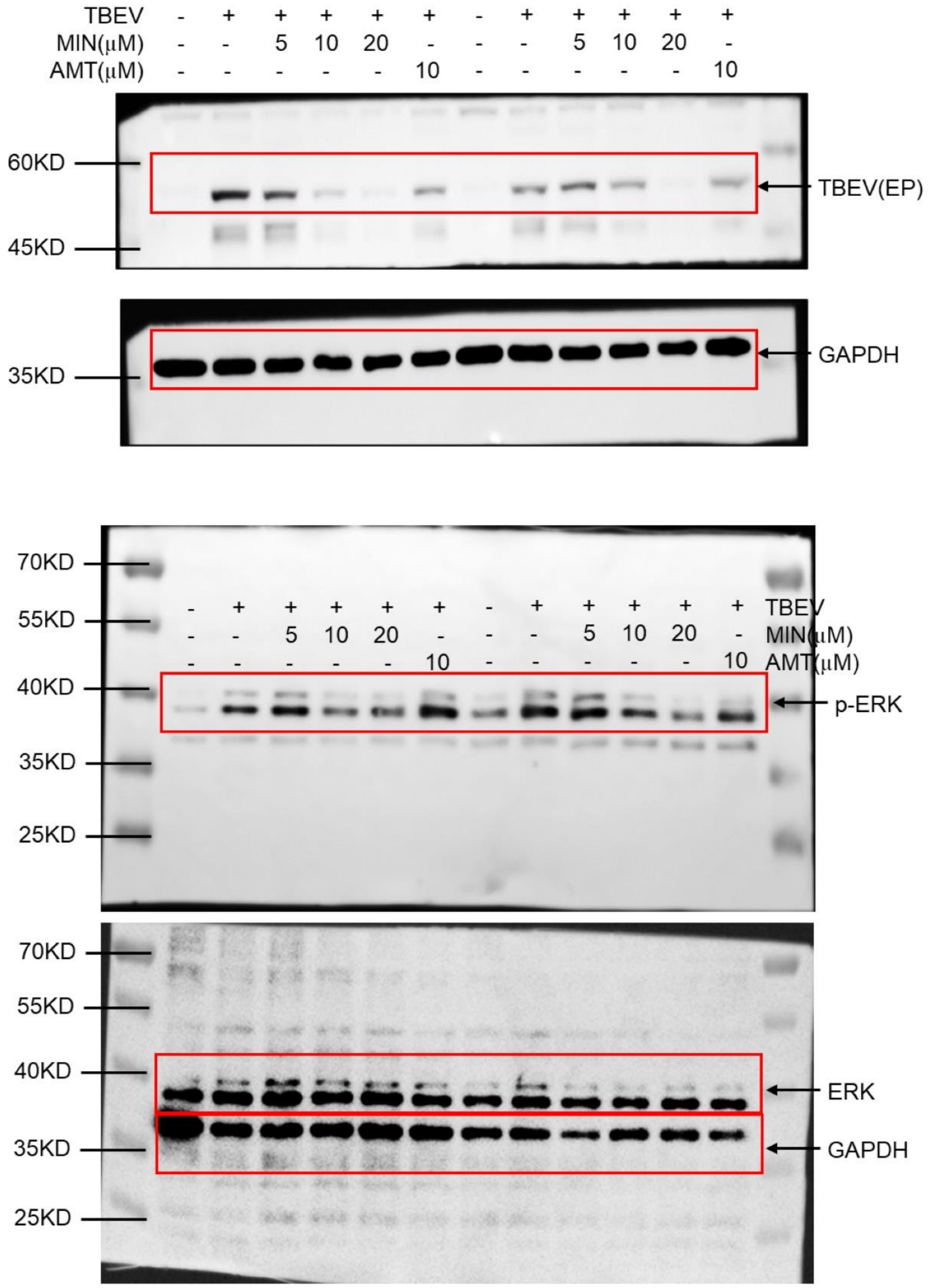

Figure S2. The Raw data of Western-blot(Figure8 C-D)

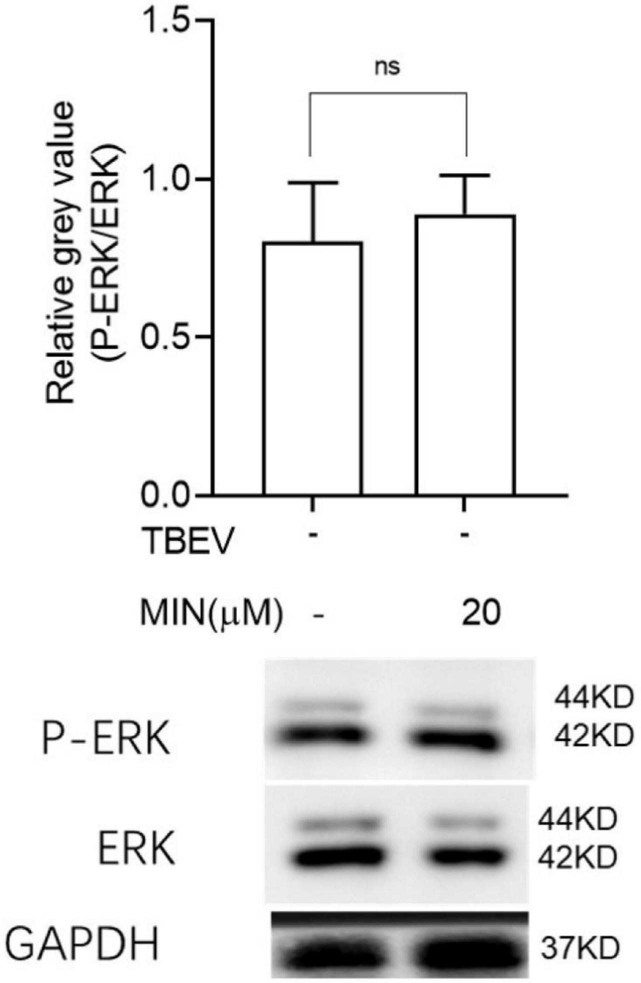

Figure S4.Western blot analysis of P-ERK/ERK expression in 20 μM MIN group compared with control group

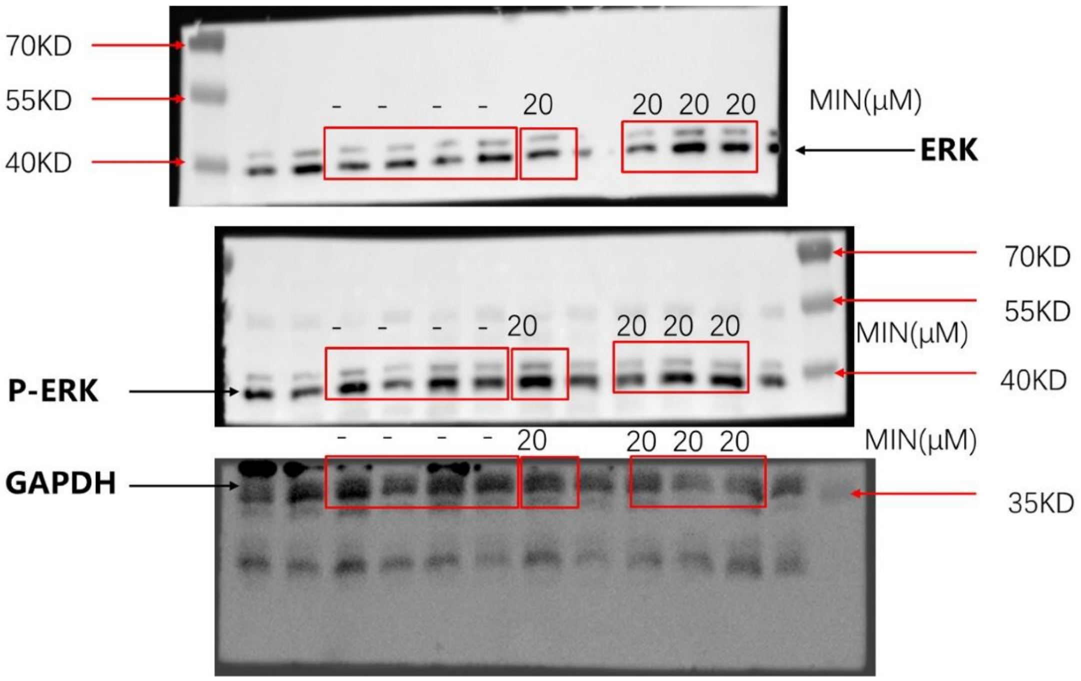

Figure S4. The Raw data of Western-blot (FigureS3)
