# Supplementary figures and images for: Minocycline Inhibits Tick-Borne Encephalitis Virus and Protects Infected Cells via Multiple Pathways
Source: Viruses. 2024 Jun 29;16(7):1055. doi: 10.3390/v16071055 (PMC11281541; doi:10.3390/v16071055)

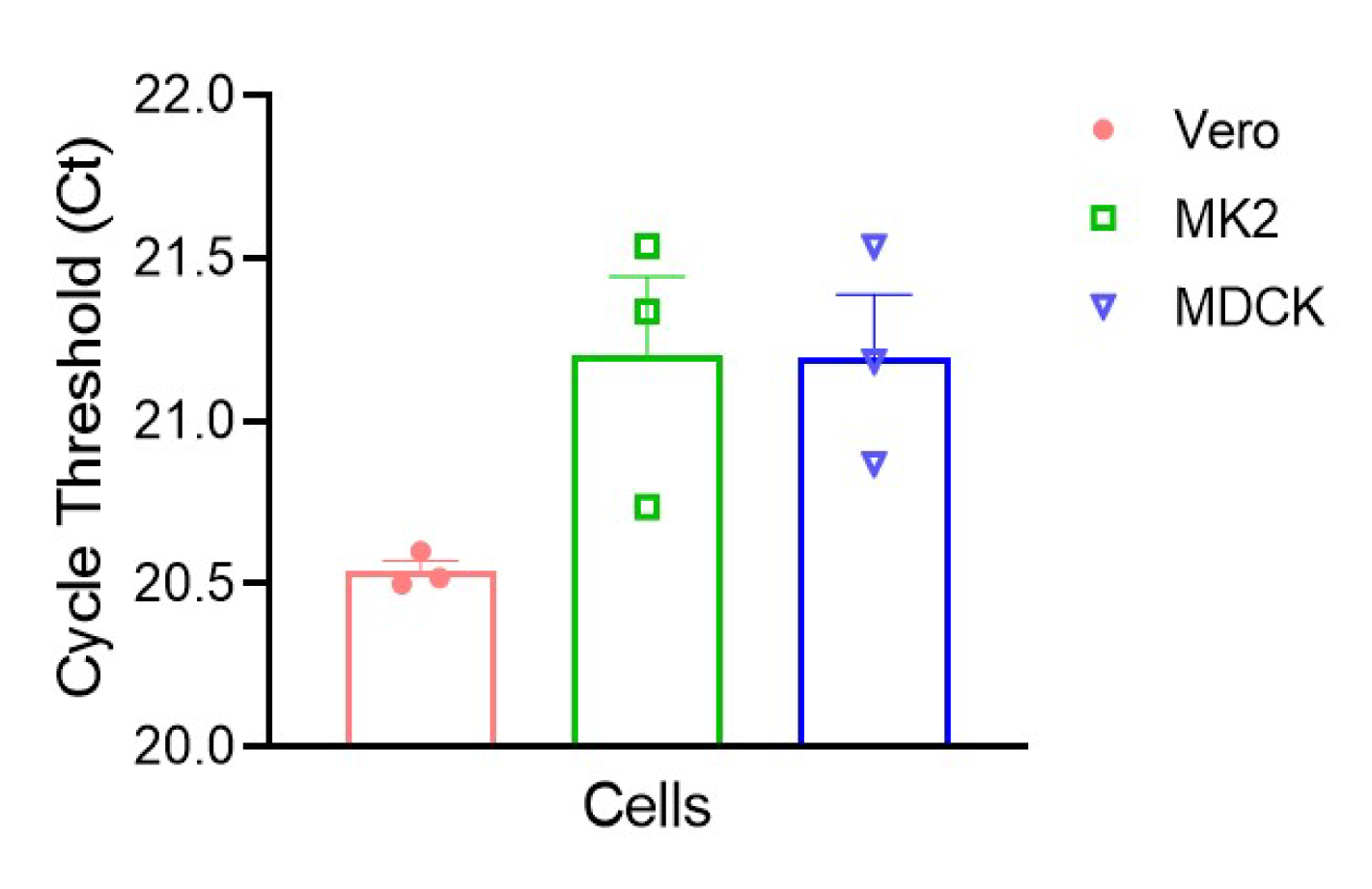

Supplement: Supplementary file 1 [file viruses-16-01055-s001.zip › Fig S1.tif]

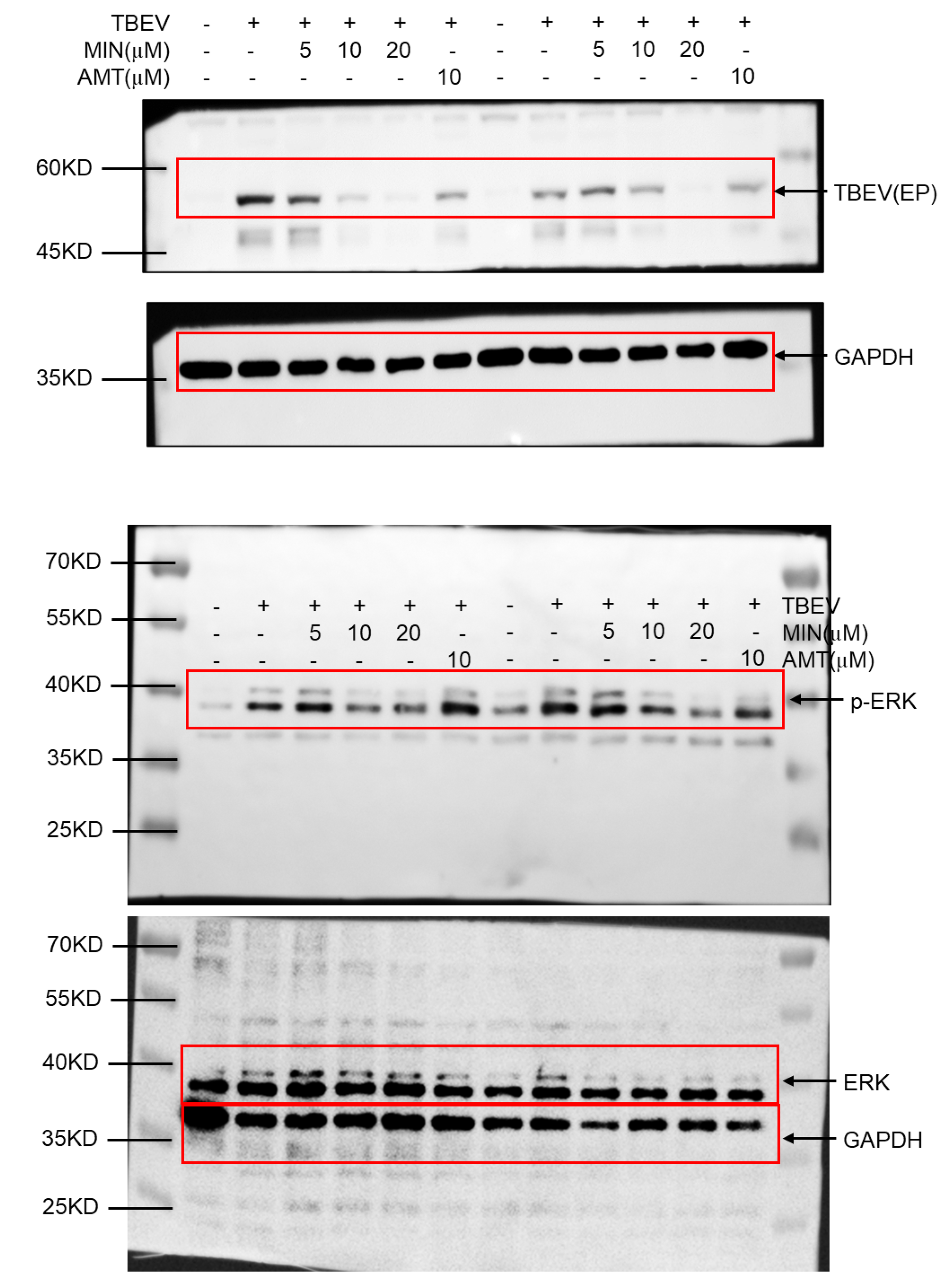

Supplement: Supplementary file 1 [file viruses-16-01055-s001.zip › Fig S2.tif]

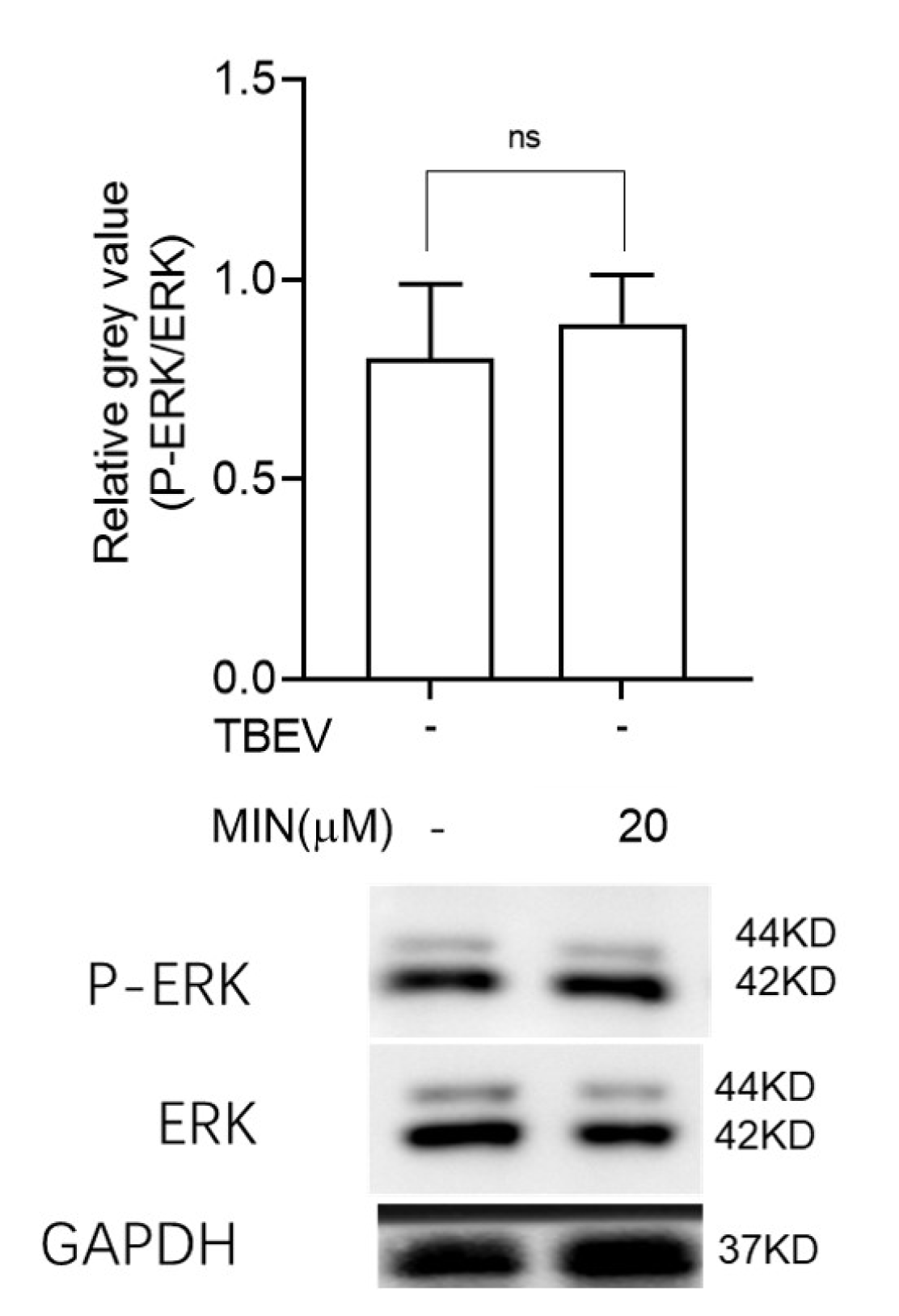

Supplement: Supplementary file 1 [file viruses-16-01055-s001.zip › fig S3.tif]

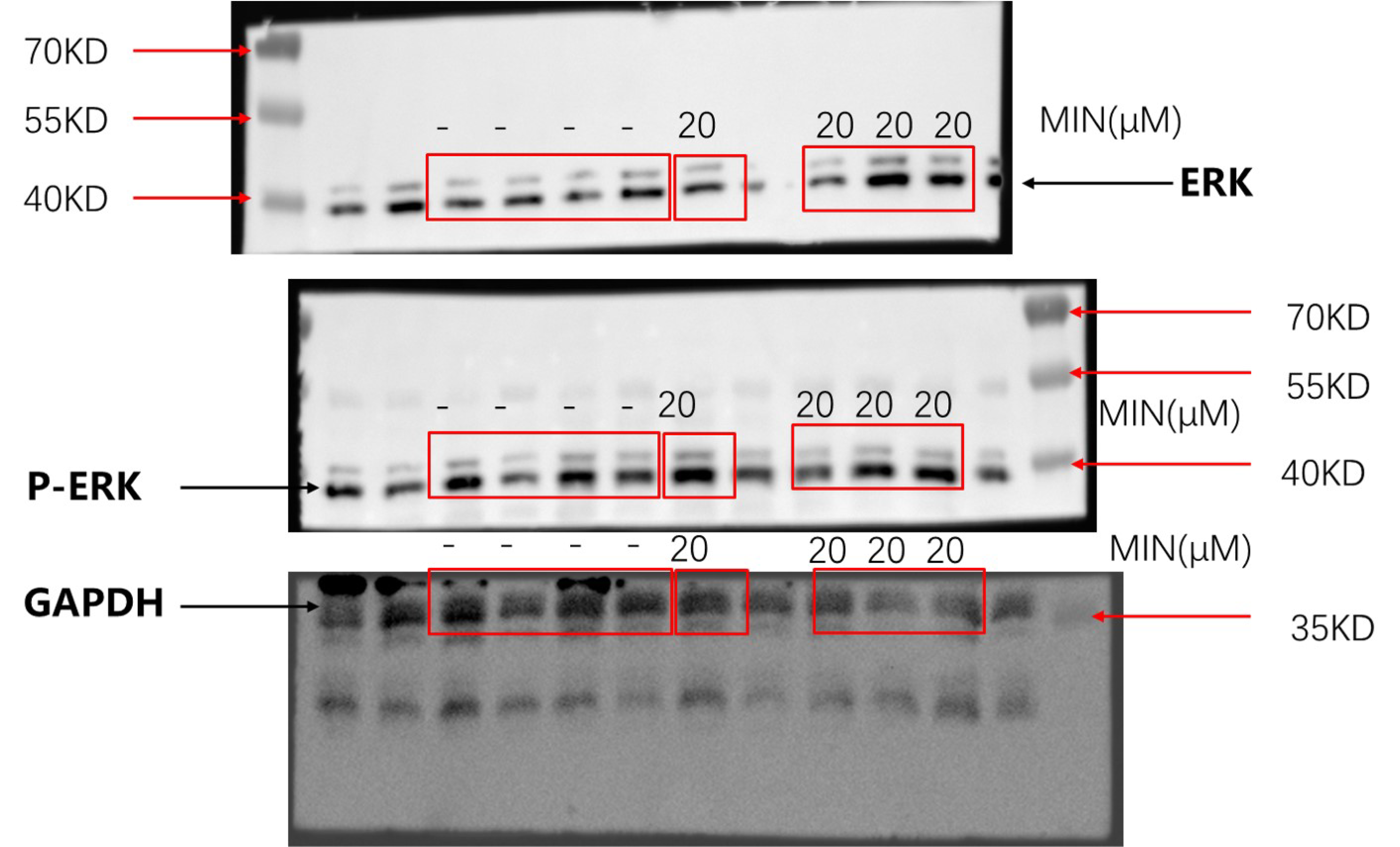

Supplement: Supplementary file 1 [file viruses-16-01055-s001.zip › fig S4.tif]
